# Is the Medical Oncology Workforce in Canada in Jeopardy? Findings from the Canadian Association of Medical Oncologists’ COVID-19 Impact Survey Series

**DOI:** 10.3390/curroncol31080319

**Published:** 2024-07-27

**Authors:** Lauren Jones, Bruce Colwell, Desiree Hao, Stephen Welch, Alexi Campbell, Sharlene Gill

**Affiliations:** 1 Division of Medical Oncology, BC Cancer, University of British Columbia, Vancouver, BC V5Z 4E6, Canada; 2 Division of Medical Oncology, Queen Elizabeth II Health Sciences Center, Dalhousie University, Halifax, NS B3H 2Y9, Canada; 3 Department of Medical Oncology, Tom Baker Cancer Centre, Cumming School of Medicine, University of Calgary, Calgary, AB T2N 4N2, Canada; 4 Division of Medical Oncology, London Health Sciences Centre, Western University, London, ON N6A 5W9, Canada; 5 Canadian Association of Medical Oncology, Ottawa, ON K1E 3R9, Canada

**Keywords:** burnout, workforce sustainability, medical oncology, COVID-19

## Abstract

The COVID-19 (C19) pandemic introduced challenges in all areas of the Canadian healthcare system. Along with adaptations to clinical care environments, there was increasing concern about physician burnout during this time. The Canadian Association of Medical Oncologists (CAMO) has examined the effects of the pandemic on the medical oncology (MO) workforce. A series of four multiple choice web-based surveys distributed to MOs who were identified using the Royal College of Physicians and Surgeons directory and CAMO membership in May 2020 (S1), July 2020 (S2), December 2020 (S3), and March 2022 (S4). Descriptive analyses were performed for each survey, and a Chi-square test (α = 0.05) was used to assess factors associated with planned change in practice in S4. The majority of respondents work in a comprehensive cancer center S1/S2/S3/S4 (87%/86%81%/88%) and have been in practice >10 years (56%/61%/50%/64%). The most commonly reported personal challenges were physical (60%) and mental (60%) wellness. In S4, 47% of MOs reported dissatisfaction with their current work–life balance. In total, 83% reported that their workload has increased since the beginning of C19, and 51% of MOs reported their future career plans have been impacted by C19. In total, 56% of respondents are considering retiring or reducing total working hours in the next 5 years. Since the onset of the C19 pandemic, there are concerns identified with wellness, increasing workload, and job dissatisfaction among MOs, associated with experienced staff who have >10 years in practice. As rates of cancer prevalence rise and treatments become more complex, it is crucial to address the concerns raised in these surveys to ensure that we have a stable MO workforce in the future.

## 1. Introduction

The World Health Organization declared the global COVID-19 (C19) pandemic in March 2020. Healthcare systems needed to adapt rapidly to a changing environment amidst concerns about C19 exposure, impact on patient care, and personal wellness. There is evidence of increasing physician burnout since the onset of the C19 pandemic, with more than half of all physicians reporting burnout, with nearly half also considering reducing their clinical working hours in the next 2 years [1]. In a global survey series, the European Society of Medical Oncologists (ESMO) Resilience Task Force also identified that 38% of medical oncologists (MOs) are considering leaving oncology entirely [1].

Concerns about burnout and wellbeing among oncologists have been present since before the C19 pandemic. Banerjee et al. found that 71% of oncologists across 41 countries aged 40 or younger showed evidence of burnout in 2017 [2]. Singh et al. showed that 73% of cancer care physicians in Ontario had symptoms of burnout in 2019, and at that time, 49% were considering reducing working hours and 25% were considering retiring or changing careers [3]. Dahn et al. surveyed Canadian oncology residents in 2019 and found that 42% met the defined burnout criteria [4]. Shanafelt et al. reported that 45% of oncologists were burned out in a US-based survey in 2012 [5].

Increasing workload is another concern for MOs. Nearly half of Canadians (1 in 2.2) are expected to develop cancer, with incidence increasing with age. Canada has a growing and aging population in which the prevalence of cancer among Canadians continues to rise [6]. Survivorship is also increasing over time, likely due to screening, early detection, and improved treatment options for many types of cancers; five-year net survival has increased by 9% between 1992 and 1994 and 2015 and 2017 [6] with projections expected to increase further in the next decade [7,8]. The increasing incidence, prevalence, and complexity of cancer care requires a thoughtful examination of the MO workforce and its resilience in the face of increasing demands.

The C19 pandemic has introduced new challenges, and exacerbated pre-existing challenges in the Canadian healthcare system. The Canadian Association of Medical Oncologists (CAMO) has been examining the effects of C19 on the MO community, which may have provided further stress to a workforce with known high burnout prevalence. This has been evaluated through a four-part survey series. The first three surveys focused on the risk of personal COVID exposure, testing practices, concerns about the impact care of oncology patients, use of telemedicine, and personal wellness [9,10,11]. The findings from the first survey were previously reported [11], and this article describes the additional findings from survey 2 (S2), survey 3 (S3), and survey 4 (S4).

The final survey in the series (S4) was distributed at the time when C19 shifted from a pandemic toward an endemic [12]. The objective was to assess longitudinal trends, gauge wellness among Canadian MOs, and assess workforce trends that may impact MO workforce capacity going forward. We hypothesized that concerns regarding wellness and workload would persist beyond the acute phase of the C19 pandemic.

## 2. Methods

A series of multiple choice web-based surveys were distributed via SurveyMonkey (SurveyMonkey, San Mateo, CA, USA), to MOs using convenience sampling through the CAMO membership directory and those who were identified via the Royal College of Physicians and Surgeons directory. Participation was voluntary. Survey results were anonymous and therefore were not tracked longitudinally among prior participants. Surveys were distributed in May 2020 (S1), July 2020 (S2), December 2020 (S3), and March 2022 (S4). S1, S2, and S3 included domains about personal wellness, risk of oncologist exposure and diagnosis of C19, patient risk of C19 diagnosis, impact on use of telemedicine, clinical trial accrual, concerns about adequate access to PPE for employees, and access to adequate healthcare for patients. S4 was distributed after acute waves of the C19 pandemic. This survey continued to investigate personal wellness trends, and introduced new questions to assess workload, workplace resources, and planned change in practice. Descriptive analyses were performed for each survey, and a Chi-square test (α = 0.05) and stepwise linear multiple regression were performed (SPSS V29.0.2.0) to assess factors associated with planned changes in practice. 

## 3. Results

### 3.1. Baseline Characteristics

Table 1 presents the baseline characteristics across all four CAMO surveys. S1–S3 were sent to 323 emails, and S4 was sent to 477 emails. Response rate for survey 4 (S4) was 32% (*n* = 151/477). In total, 66% of respondents completed at least one prior survey, and 21% completed all three prior surveys. In total, 88% of respondents practiced in comprehensive cancer centers, and 46% were in practice for more than 15 years. At least 60% of respondents in all surveys are CAMO members. 

### 3.2. COVID-19 Exposure Risk and Impact on Patient Care

S1/S2/S3 demonstrated that levels of moderate/extreme concerns about personally catching C19 (79%/54%/59%) and concerns about patients catching C19 (71%/48%/73%) fluctuated throughout the pandemic. Confidence in adequate healthcare access increased between S1/S2 (39%/59%), and clinical trial accrual also increased (46%/67%). The use of telemedicine decreased from S1 in 2020 (45%) to S4 in 2022 (5%). 

### 3.3. Personal Wellness

**Figure 1:**
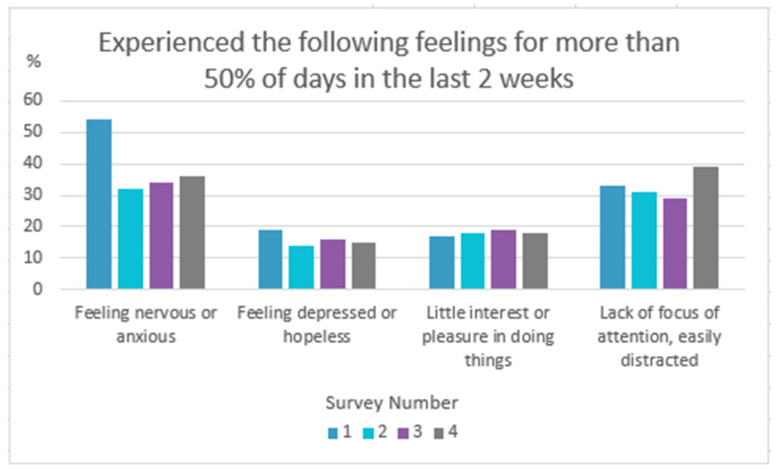
Personal wellness trends throughout the C19 pandemic.

Physical (60%) and mental (60%) wellness were reported as the biggest personal challenges in S4. Domains of personal wellness including feeling nervous or anxious, feeling depressed or hopeless, little interest/pleasure in doing things, and lack of focus/feeling easily distracted were assessed through all four surveys. The feeling of being easily distracted peaked in S4 (39%), while feeling anxious and feeling depressed relatively decreased since the beginning of the pandemic (Figure 1). 

### 3.4. Workload

In S4, 47% of MOs reported dissatisfaction with their current work–life balance. In total, 83% reported that their workload increased since the beginning of C19. Workload domains were assessed by asking about challenges that occur on more than 50% of days in the past 12 months, including longer working hours (61%), inadequate personal time (55%), inability to take breaks during work (64%), inability to take vacation (40%), and increased after hours work (59%). In total, 62% of respondents did not feel that their workplace has the resources to help support a changing scope of practice in the future, and only 27% of MOs feel at least moderately valued by their workplace.

### 3.5. Change in Practice

In S4, 51% of MOs reported that their future career plans have been impacted by C19. In total, 56% of respondents are considering retiring or reducing total working hours in the next 5 years. In total, 35% have considered leaving MO entirely. In total, 48% of MOs would prefer to have less clinical work involving direct or indirect patient care. Career length >10 years and age >40 was associated with considering leaving MO (*p* = 0.01 and *p* = 0.03 respectively). Career length >10 years was associated with the consideration of reducing total working hours within the next 5 years (*p* = 0.045) (Table 2). No significant independent associations were observed using multiple regression.

## 4. Discussion

There have been various challenges presented throughout the duration of the C19 pandemic, and temporal trends have emerged from this series of surveys. Concerns about personal C19 risk were highest in S1 and S3, corresponding with timing of the first two “waves” of the pandemic.

Telemedicine use increased substantially at the start of the pandemic, reflecting the ability of the MO workforce to rapidly adapt changes in provision of care to ensure that there was minimal disruption for cancer care. In S1, there was concern about adequate healthcare access and decreased clinical trial accrual, which improved in S2 as the pandemic continued and healthcare provision and clinical trial protocols adapted in response to a changing healthcare environment. 

The changing scope of medicine and the realities of an overburdened healthcare system existed before the C19 pandemic. In 2017, the CMA National Physician Health Survey identified that 30% of respondents across all medical specialties had high burnout [13]. A survey of 36,000 primary healthcare clinicians in the US was distributed in March 2022 and revealed that 1 in 5 primary care physicians intend to leave practice [14]. The C19 pandemic presented an acute stress on a healthcare system experiencing concerns with workload and burnout, bringing these concerns to the forefront and highlighting the importance of adapting and restructuring the way in which healthcare and medical oncology services are delivered in the future. This survey series reviewed the experience of medical oncologists to gain a deeper understanding of the specialty-specific impacts of C19 and understand how the future of oncology care may shift. 

It has been reported that factors associated with burnout among oncologists include young age, early career, and female oncologists, while high psychological resilience is protective against burnout [5,13,15,16,17]. Interestingly, in our survey, we found that MOs most likely to consider leaving the career entirely were over age 40 and had over 10 years in practice. This discrepancy suggests that burnout may not be the only driving factor for Canadian MOs who are considering leaving MO, and warrants further investigation. Addressing burnout among MOs is critical during pandemic times and beyond. Hlubocky et al. identified clinical, personal, and external variables that contribute to moral distress and how they were impacted by C19, and they also developed a framework for oncologist well-being throughout the various phases of a pandemic, which highlight the importance of interventions such as education, leadership involvement, stress management, therapeutic, and grief supports [18]. These recommendations can be extrapolated to apply to future pandemics and times of crisis in healthcare.

There is evidence of increasing and changing MO workload in Canada. Before the C19 pandemic, it was reported that half of MOs in Canada were exceeding the yearly recommended target of 160–175 new patient consultations [19]. This suggests an unsustainable workload considering that because of the progressive complexities and improvements in oncological treatments, patients require longer-term follow up compared to the early 2000s, when this benchmark was established. In a study of all forms of patient care encounters based in Nova Scotia, Canada, there was a 9.5% increase in encounters, including consultations, follow up visits, telephone visits, and chart checks, per year between 2014 and 2022, with a 46% increase in virtual care encounters between 2019 and 2020 [20]. Pre-existing high workload exceeding national targets before the C19 pandemic paired with rising cancer incidence raises concerns about inadequate numbers of MOs to treat patients going forward.

Pre-pandemic, the Canadian MO workforce had 1.7 physicians per 100,000 population; 62% of MOs were age 45 or older, and 64% reported being satisfied with their professional life [21]. In a study by Fundytus et al., it was found that in 1994, 24% of MOs were age 50 or older; this has increased to 40% in 2020 [22]. This highlights the importance of a recruitment strategy to sustain cancer care. As senior oncologists begin planning for retirement, especially in light of findings of high numbers of MOs considering retiring early following the C19 pandemic.

Perhaps the most concerning finding is that 35% of respondents are considering leaving MO practice entirely, which is consistent with findings from an ESMO global survey series [1]. The Great Resignation is a term coined to describe the workforce exodus in the wake of C19, and healthcare is no exception [23]. Exploring reasons for work dissatisfaction, and implementing solutions for concerns including, but not limited to, workload demands and burnout is necessary to retain MO workforce capacity and expertise in Canada. There is an urgent need to address the issue of workforce crisis globally, in 2024, a ‘Cancer Workforce Fund’ was announced in Europe and the UK to fund innovation and identify possible solutions to maintain a robust cancer care workforce [24]. Initiatives like these will be important to develop in Canada to tackle this growing concern to sustain the MO workforce by supporting workers in all disciplines of cancer care.

Limitations of this survey series include a low response rate across all surveys, anonymous responses, and the development of follow up surveys throughout the C19 pandemic which limited capacity to link responses of participants in serial surveys. There is also the potential for bias that respondents who are more frustrated with their career may feel more strongly about completing the survey. 

## 5. Conclusions

MO concerns about C19 infections, personally and for patients, have decreased since the start of the pandemic. Clinical tools such as telemedicine were implemented in high proportions initially, and the use of telemedicine as a clinical tool continued through the pandemic and will likely continue to be an important component of cancer care in the future.

As we move beyond the challenges faced at the onset of the C19 pandemic, which highlighted the need for more robust crisis preparedness plans in healthcare for future pandemics or system stressors, there are also concerns identified with wellness, workload escalation, and job dissatisfaction among MOs. One-third of respondents report considering leaving MO practice, and this was associated with >10 years in practice, which suggests the potential loss of the senior, experienced workforce. In the face of escalating demand for MO services with rising cancer incidence, prevalence, and treatment complexity, proactive strategies targeting recruitment, wellness, workload management, and retention are needed to ensure the stability of Canadian cancer care delivery going forward. CAMO has since assembled a Task Force on Medical Oncologist Wellness, Sustainability, and Burnout to investigate and address this issue, and plans are underway to develop recommendations to strengthen and stabilize the Canadian MO workforce. 

**This study has been presented in parts in:**

- Gill S, Hao D, Hirte H, Campbell A, Colwell B. ‘Impact of COVID-19 on Canadian medical oncologists and cancer care: Canadian Association of Medical Oncologists survey report’. *Curr Oncol*. 2020;27(2):71–74. https://doi.org/10.3747/CO.27.6643.
- Gill S, Colwell B, Hirte H, Stephen W, Campbell A, Hao D. Abstract PO-016: ‘Evaluating the impact of COVID-19 on medical oncology workforce and cancer care in Canada: A serial survey study’. *Clinical Cancer Research*. 2020;26(18_Supplement):PO-016. https://doi.org/10.1158/1557-3265.COVID-19-PO-016.
- Gill S, Colwell B, Welch S, Hao D. 1606P Impact of COVID-19 (SARS-CoV-2, C19) on medical oncologists (MOs) and cancer care: A Canadian Association of Medical Oncologists (CAMO) survey study. *Annals of Oncology*. 2021;32:S1148. https://doi.org/10.1016/j.annonc.2021.08.1599.
- Jones L, Colwell B, Hao D, Welch S, Campbell A, Gill S. 505P The impact of COVID-19 on the wellness and resilience of the Canadian medical oncology workforce: A Canadian Association of Medical Oncologists survey. *Annals of Oncology*. 2022;33:S774. https://doi.org/10.1016/j.annonc.2022.07.633.
- Jones, L., Colwell, B., Hao, D., Welch, S., Campbell, A., Gill, S. The Impact of COVID-19 on the Wellness and Resilience of the Canadian Medical Oncology Workforce: A Canadian Association of Medical Oncologists Survey Study, CAMO Annual Scientific Meeting, April 2023.

## Figures and Tables

**Table 1:** Baseline characteristics.

	Survey 1 *n* = 159	Survey 2 *n* = 157	Survey 3 *n* = 124	Survey 4 *n* = 151
Survey date	May 2020	July 2020	Dec 2020	March 2022
Practice setting				
Comprehensive Cancer Center	87%	86%	81%	88%
Community Practice—Urban	11%	12%	16%	11%
Community Practice—Rural	2%	2%	2%	1%
Years in practice				
<5 years	25%	22%	26%	19%
5–10 years	19%	17%	24%	18%
10–15 years	15%	15%	11%	18%
>15 years	41%	46%	39%	46%
Province				
British Columbia	26%	19%	23%	31%
Alberta	24%	13%	15%	14%
Saskatchewan/Manitoba	9%	8%	8%	9%
Ontario	28%	38%	34%	31%
Quebec	6%	14%	8%	5%
Atlantic *	7%	8%	12%	9%
CAMO Member	60%	65%	67%	63%

* Newfoundland, New Brunswick, Nova Scotia, Prince Edward Island.

**Table 2:** Factors associated with planned change in practice [12].

	*n*	Considering Leaving Medical Oncology		Considering Reducing Hours/FTE	
Gender					
Female	88	53%	*p* = 0.23	59%	*p* = 0.69
Male	57	45%		40%	
Age					
<40	32	12%	*p* = 0.03	20%	*p* = 0.43
>40	114	88%		80%	
Practice setting					
Comprehensive cancer center	130	94%	*p* = 0.08	89%	*p* = 0.58
Other	18	6%		11%	
Years in practice					
<10	55	23%	*p* = 0.01	30%	*p* = 0.045
>10	93	77%		70%	
Feel valued by institution					
Yes	40	27%	*p* = 0.98	24%	*p* = 0.36
No	108	73%		76%	
Feel valued by public					
Yes	60	38%	*p* = 0.70	45%	*p* = 0.26
No	88	62%		55%	

## Data Availability

The data presented in this study is available on request from the corresponding author.
